# European Human Rights Dimension of the Online Access to Cultural Heritage in Times of the COVID-19 Outbreak

**DOI:** 10.1007/s11196-020-09712-x

**Published:** 2020-05-04

**Authors:** Elżbieta Kużelewska, Mariusz Tomaszuk

**Affiliations:** Faculty of Law, University of Bialystok, Ul. Mickiewicza 1, 15-213 Białystok, Poland

**Keywords:** Right to culture, Cultural heritage, Human rights, European Court of Human Rights, COVID-19

## Abstract

The 1948 Universal Declaration of Human Rights recognized that “everyone has the right freely to participate in the cultural life of the community, to enjoy the arts and to share in scientific advancement and its benefits”. As a result, cultural rights have been understood as inseparable from human rights and require protection mechanisms within particular international (including regional) legal systems. The European continent is proud to have developed one of the most effective mechanisms of the human rights protection by establishing the Council of Europe and adopting the European Court of Human Rights. The recent outbreak of the COVID-19 reformulated many concepts of access to human rights and possibilities to enjoy freedoms. Even if access to culture (access to cultural heritage) has been available online for many years, it is the time of globally occurring lockdowns that forced people to stay home and found themselves in a situation when all of a sudden online access to culture became the only way of access to culture. The article aims to analyze the current situation in Europe by asking questions if and how online access to culture is recognized and protected under the Council of Europe’s mechanisms with special emphasis on the case-law of the European Court of Human Rights in this field.

## Introduction

Access to culture (understood also as access to cultural heritage) is one of the human rights recognized through the interpretation of international and European documents including the 1948 Universal Declaration of Human Rights, the 1966 International Covenant on Economic, Social and Cultural Rights, the 1950 European Convention on Human Rights, and 2000 Charter of Fundamental Rights of the European Union.

The current health crisis (caused by the instant spread of the Coronavirus Disease 2019—COVID-19) has reshaped the world on many levels and rapidly allowed new solutions to become an every-day online reality. Among many changes, the COVID-19 outbreak immensely influenced the culture which has become massively available for everyone having access to Internet. The universal ‘lockdowns’ closed galleries, museums, theatres and other cultural institutions. The only successful way to be involved in cultural collections is to move with activity into the web world and to create online versions of cultural activities.

The paper aims to analyze whether and why human rights are respected in terms of access to online culture. The present health crisis attests that access to online cultural heritage can be enhanced.

The scope of the paper is limited in two ways. Firstly it will address very recent issues and as such can be based on the so-far available sources. Secondly, the paper will discuss only the European dimension of the undertaken problem, as according to available data the biggest percentage of Internet users live in Europe. For the purpose of the presented article, European dimension is further understood as the sphere covering the acts and activities of the Council of Europe and the European Court of Human Rights (ECtHR). The European Union perspective is left outside the scope of the research.

Two hypothesis to be examined in this paper are the following:

1. Despite the emergency situation, in which many human rights (in particular civil ones) are restricted, human rights in access to culture are not violated; they are transferred to another (online) dimension. This, in turn, may become a contribution to more effective human rights’ protection in the future;
2. Online access to culture is recognized by the European Court of Human Rights as human rights and is protected by the Convention for the Protection of Human Rights and Fundamental Freedoms (commonly known as the European Convention on Human Right).

The paper is divided into three parts.

- Part I provides general description of the access to culture as one of the human rights in the light of the fundamental European human rights documents.
- The second part is devoted to the European Court of Human Rights’ case-law addressing the online access to culture.
- The last part analyzes the cultural heritage access in times of health crisis caused by the COVID-19 in Europe.

The analysis of statistical data, relevant legal acts as well as the European Court of Human Rights’ case-law have been used in this paper as basic research methods.

## Access to Culture/Cultural Heritage as a Human Right

For the purpose of this article, the authors decided to use the terms “culture” and “cultural heritage” interchangeably, as synonyms. According to the definition of cultural heritage provided by the UNESCO, the term includes several main categories of heritage: cultural heritage sensu stricto including tangible cultural heritage (movable cultural heritage, immovable cultural heritage and underwater cultural heritage), intangible cultural heritage (oral traditions, performing arts, rituals), natural heritage and heritage in the event of an armed conflicted. UNESCO defines culture as the set of distinctive spiritual, material, intellectual and emotional features of society or a social group, that encompasses, not only art and literature, but lifestyles, ways of living together, value systems, traditions and beliefs [27, p. 9]. Therefore, the culture as understood by UNESCO is included into the definition of cultural heritage (as both tangible and intangible cultural heritage).

The cultural heritage includes all cultural goods associated with a „heritage value” [23, p. XI]. The exhaustive evolution of this phenomenon is presented by Vecco [30, p. 324]. Cultural heritage has been progressively expanded to include historic and artistic values, the cultural value, its value of identity and the capacity of the object to interact with memory. In broad sense cultural heritage encloses rich and valuable touchable objects and materials gathered in the collections by cultural institutions [3, p. XIX].

The 1948 Universal Declaration of Human Rights [29] in art. 27 indirectly expresses the right to cultural heritage and its protection: “1. Everyone has the right freely to participate in the cultural life of the community, to enjoy the arts and to share in scientific advancement and its benefits; 2. Everyone has the right to the protection of the moral and material interests resulting from any scientific, literary or artistic production of which he is the author”. This article reflects the idea that culture carries an universal aspect of the human rights concept [26, p. 4].

The 1966 International Covenant on Economic, Social and Cultural Rights in art. 15 guarantees everyone the right to participate in cultural life, to enjoy the benefits of scientific progress and its applications, and to benefit from the protection of the moral and material interests resulting from any scientific, literary or artistic production of which he is the author. The States Parties to the Covenant are obliged to take steps to “to achieve the full realization of this right shall include those necessary for the conservation, the development and the diffusion of science and culture” [10].

On 6 October 2016, the Human Rights Council of the United Nations (UN) unanimously adopted a resolution calling upon all States to respect, promote and protect the right of everyone to take part in cultural life, including the ability to access and enjoy cultural heritage [25]. The UN Special Rapporteur stressed the rights to access to, and enjoyment of cultural heritage [28].

The above documents are examples of the universal acts adopted within the United Nations [1, pp. 32–33]. In Europe, the human rights protection efforts have been most extensively carried out within the Council of Europe—the continent’s leading human rights organization with 47 member states (including all the member state of the European Union). Human rights protection system developed by the Council of Europe includes the European Convention on Human Rights and Fundamental Freedoms and the European Court of Human Rights. The system is proved to be the most effective international mechanism of human rights protection in the world [15, p. 45; 16, p. 80; 20, p. 221].

Adopted in 1950 the European Convention on Human Rights and Fundamental Freedoms (Convention) does not directly provide for the right to culture or the right to take a part in cultural life. However, the European Court of Human Rights’ case-law includes examples of the protection of certain rights which are in fact the right to culture and are reconstituted from other rights, in particular freedom of speech (art. 10), right to respect for private and family life (art. 8) and right to education (art. 2 Protocol No. 1).

The fundamental European document addressed to human rights protection (including right to culture/culture heritage) is the Convention, however, there are other documents devoted to culture protection, such as the 1954 European Cultural Convention which aims to protect the common cultural heritage and to stimulate cultural development in the member states of the Council of Europe [7]. Other specific countries’ tasks and methods of their support with regard to the protection of cultural heritage are provided in the subsequent Council of Europe’s conventions that is the 1985 Convention for the Protection of the Architecture Heritage of Europe and the 2001 European Convention for the Protection of the Audiovisual Heritage. The key recent document is the 1990 (adopted in 2012) Recommendation on the right of everyone to take part in cultural life [24] as it recommends equal right and free access to cultural resources of all kinds for all, an obligation on Member States’ public authorities to ensure a broad supply of cultural services through all its public institutions. The State also has a duty to take account of radical changes in the ways in which it accesses culture, given the rapid development of digital culture and the Internet, to facilitate the emergence of new artists and new forms of expression, and to develop new ways of disseminating culture in order to make it accessible to all [21, p. 34].

The cultural heritage is important especially in relations to human dimension. The right of access to and enjoyment of cultural heritage is a legal basis in various human rights norms. This perfectly shows that cultural heritage is world widely recognized as one of the fundamental human rights. Such attitude is reflected in the European approach to the cultural rights protection.

Internet is seen as an invaluable space for the exercise of fundamental rights such as freedom of expression and information [13, p. 172]. Thus the Council of Europe’s Committee of Ministers recommends that “opportunities arising from the new digital environment should be used to reinforce access to and participation in open culture, thereby strengthening democracy”.^1^

However, Internet’s potential to reinforce access to culture and strengthen democracy is mediated by many challenges. “Such challenges include the creation of echo chambers; concerns about privacy, ownership, piracy, misinformation and interference by governments; and anonymity that may provide useful cover for both activists and criminals, as well as an apparent license for incivility. In addition, though some 85% of European households have access to the internet, not everyone has equal access or the capacity to participate equally in online cultural and political life” [19, p. 7].

Jasmontaite and de Hert rightfully state that access to the Internet should be recognized as a human right because it is involved in present life and it has received the status of a ‘driving force’ of personal, social and economic growth [14, p. 172]. If we agree that Internet is a space for the exercise of human rights and further that the Internet access facilitates the exercise of various rights, it means that online access to culture is one of the human rights and deserves to be protected. Hence, digital rights are human rights [8, pp. 8–17].

## The European Court of Human Rights’ Case-Law on Access to Culture

The problems of accessibility to the cultural heritage via Internet and the role of the Internet itself in the modern world have been raised few times in the history of the European Court of Human Rights’ jurisdiction [5] and they will be shortly presented below. However, the number cases concerning violations of the spectrum of rights referring to access to culture in general is impressive as shown in the Table 1.

**Table 1:** Number of applicants and judgments regarding the sphere of access to culture. *Source*: ECtHR https://echr.coe.int/Pages/home.aspx?p=caselaw&c=#n14597620384884950241259_pointer. Accessed 20 April 2020

Articles of the ECHR	Number of Applications by State	Total number of judgments
art. 8	Albania (11), Andorra (9), Armenia (36), Austria (262), Azerbaijan (44), Belgium (103), Bosnia and Herzegovina (17), Bulgaria (388), Croatia (270), Cyprus (32), Czech Republic (133), Denmark (57), Estonia (69), Finland (199), France (575), Georgia (30), Germany (418), Greece (153), Hungary (169), Iceland (20), Ireland (61), Italy (595), Latvia (144), Lithuania (149), Luxembourg (22), Malta (41), Republic of Moldova (140), Montenegro (18), Netherlands (164), North Macedonia (64), Norway (104), Poland (462), Portugal (114), Romania (470), Russia (700), San Marino (10), Serbia (73), Slovakia (120), Slovenia (133), Spain (141), Sweden (139), Switzerland (377), Turkey (732), Ukraine (288), United Kingdom (710)	8915
art. 9	Armenia (30), Austria (31), Azerbaijan (5), Belgium (11), Bosnia and Herzegovina (10), Bulgaria (57), Croatia (5), Denmark (8), France (77), Georgia (12), Germany (23), Greece (98), Hungary (36), Iceland (5), Ireland (5), Italy (40), Latvia (9), Lithuania (7), Republic of Moldova (32), Netherlands (4), North Macedonia (12), Poland (13), Romania (40), Russia (67), San Marino (3), Slovakia (4), Spain (25), Sweden (8), Switzerland (9), Turkey (188), Ukraine (19), United Kingdom (69)	950
art. 10	Armenia (17), Austria (228), Azerbaijan (42), Belgium (54), Bosnia and Herzegovina (16), Bulgaria (85), Croatia (23), Cyprus (22), Czech Republic (13), Denmark (30), Estonia (56), Finland (118), France (297), Georgia (19), Germany (175), Greece (105), Hungary (208), Iceland (54), Ireland (21), Italy (125), Latvia (51), Liechtenstein (5), Lithuania (49), Luxembourg (17), Malta (17), Republic of Moldova (147), Montenegro (5), Netherlands (54), North Macedonia (14), Norway (49), Poland (206), Portugal (97), Romania (154), Russia (233), Serbia (36), Slovakia (37), Slovenia (14), Spain (129), Sweden (39), Switzerland (227), Turkey (1250), Ukraine (102), United Kingdom (232)	4869
art. 2 Protocol No. 1	Austria (5), Belgium (6), Bulgaria (42), Croatia (19), Czech Republic (31), Denmark (8), Greece (36), Hungary (8), Italy (69), Republic of Moldova (27), Norway (25), Romania (4), Russia (56), Sweden (13), Turkey (78), Ukraine (2), United Kingdom (14)	416

The starting point for the discussion on the online access to culture is the 2009 ruling in *Times Newspapers Ltd. v. The United Kingdom* In this ruling the ECtHR confirmed that the Internet plays an important role in enhancing the public’s access to news and facilitating the sharing and dissemination of information generally. It is so because of its accessibility and capacity to store and communicate vast amounts of information. The concept of giving the Internet such role in the societies’ life is the first step to confirm the importance of this medium in brokering between the receiver and the object.

Moreover, in 2010 case *Ahmet Yildirim v. Turkey* the ECtHR confirmed that the right to the Internet access cannot be limited by countries without specific statutory grounds, even though the violation of human rights may still be excused. The Court also underlined that a restriction on Internet access, as it had place in the cited case, had rendered large amounts of information inaccessible, thus substantially restricting the rights of Internet users and resulted in significant collateral effect.

It should be stressed that not all the limitations of access to specific websites are considered violation of Article 10 of the European Convention of Human Rights. As it was stated in 2010 *Akdeniz v. Turkey* case, the individual cannot claim to be a “victim” of violation of Article 10 of the Convention if he had access to searched materials (in this case music) by numerous means, excluding the Internet. Indeed the right to access the materials via one of the sources (Internet was limited), however it does not mean that he had no possibility to access it in other ways.

The ECtHR had a chance to underline the significance of specific websites providing access to culture. In the 2015 ruling *Cengiz and Others v. Turkey* the Court found that the applicants (two law professors actively using YouTube to access, download and share videos for professional purposes) could be considered as victims of the alleged breach of Article 10 as they all had YouTube accounts and made substantial use of its services for professional purposes. The Court also emphasized the importance of YouTube in exercising the freedom of expression, in disseminating and exchanging not only artistic and musical creations but also political ideas and information.

The ECtHR presents the position that Internet is important not only in the context of culture accessibility. In the 2008 case of *Khurshid Mustafa and Tarzibachi v. Sweden* (concerning the eviction of tenants on account of their refusal to remove a satellite dish that enabled them to receive television programs from their country of origin) the Court developed its case-law on freedom to receive information under Article 10 of the Convention. It emphasized the significance of such freedom for immigrants, who may wish to maintain contact with culture and language of their home country.. The Court pointed out that freedom to receive information does not extend only to reports of events of public concern, but covers in principle also cultural expressions, as well as pure entertainment.

The ECtHR further analyzed and presented in its rulings the issue of access to the cultural heritage (which were given a very special and *ad*-*hoc* meaning during the COVID-19 pandemic). Firstly, the Court developed its case-law on reconciling freedom of artistic expression and the protection of morals in the 2014 judgment *Akdaş v. Turkey*. This judgment was issued in the case related to the limitation in publishing a specific book considered immoral in Turkey. The Court stated that countries introducing any limits based on the requirements of morality need to take into consideration the existence, within the single state, of various cultural, religious, civil or philosophical communities. Therefore, the Court presented the concept of a “European literary heritage” and set out various criteria in this regard: the author’s international reputation; the date of the first publication; a large number of countries and languages in which publication had taken place; publication in book form and on the Internet; and publication in a prestigious collection in the author’s home country. The Court concluded that the public of a given language, could not be prevented from having access to work that is part of such heritage.

It has to noted however, that the Court judged not only in cases where cultural heritage regarded collective right, but also where it regarded individual right. The 2012 *Catholic Archdiocese of Alba Iulia v. Romania* may serve as an example. The case concerned the State’s failure, despite a Government regulation, to return to their former owner, a catholic religious community, a library and a museum of great historical and cultural importance. The Court emphasized that the State’s prolonged failure to act and the uncertainty affecting the applicant with regard to the legal status of the property claimed by it was all the more unreasonable when account was taken of the cultural and historical importance of the assets in question.

The access to cultural heritage may also have another dimension. In the 2015 case *Sargsyan v. Azerbaijan,* regarding the issue of applicant’s accessibility to his property and home located in Nagorno-Karabakh area, the Court stated that the impossibility for the applicant to have access to his home and to his relatives’ graves in Gulistan without the government taking any measures in order to address his rights or to provide him at least with compensation for the loss of their enjoyment, placed and continues to place a disproportionate burden on him and states a continuing breach of the applicant’s rights under Article 8 of the Convention.

Short analysis of the ECtHR cases may lead to interesting conclusions regarding the access to the cultural heritage online. Firstly, the ECtHR directly stated that access to culture via Internet (as well as television) is the manifestation of the human right stated in the article 10 of the European Convention on Human Rights that is the freedom of expression. Secondly, the ECtHR directly confirmed that the right of access to cultural heritage, (which may be communicated on various levels and be referred to various spheres of human life)—can be both individual and collective and, may touch public and private areas. As a result, the ECtHR established a wide approach to the problem of culture and cultural heritage availability.

## COVID-19 Versus Cultural Heritage Access in Europe

According to data collected by the International Telecommunication Union (ITU), in 2019 around 53.6% of the world population used Internet, which gives number of 4.131 million people having possibility to be online and using it [13]. Comparing to year 2008, the number of Internet users noted increase of 2.561 million or 30.5 percentage points. The biggest percentage of Internet users live in Europe (82.5% of total population).

The role of the Internet was noted by the Council of Europe in its Strategy for 2016–2019 which underlined the core value of the Internet as „an invaluable pace for the exercise of fundamental rights such as freedom of expression and information, of critical value for democracy. Its capacity to allow people to impart and exchange ideas (…) offers the potential to promote understanding and tolerance between people (…) [and] connecting their voices to the Internet (…) is important for pluralism and diversity in dialogue, and for bridging gaps between States and citizens” [11, pp. 9–12].^2^ Internet is a very important medium for culture accessibility and a platform for exchanging various ideas and thoughts. Access to culture via Internet has not been recognized as a human right as the result of violation thereof, but as the result of the intensive development of that medium its enormous influence on people’s habits and behaviors [8, pp. 6–7].

In the COVID-19 pandemic situation the culture became massively available for everyone who has access to the Internet. With cultural institutions closed due to the governmental restriction, their treasures are now open online for all the publicity, quite often free of charge, taking the right to enjoy the cultural heritage to a different level of accessibility. Freedom to celebrate culture is now linked to the Internet availability [9, p. 390]. At the same time, spreading misinformation about COVID-19 has become part of a polarized political narrative and of manipulation campaigns that may be state controlled. Some countries have used the misinformation narrative as a justification to introduce disproportionate restrictions to fundamental rights and freedoms [17, p. 369].

As the recent study conducted by Dalia Research shows, in 2017 31% of the Internet users in 28 EU countries created cultural content online, 27% discussed cultural content, 52% searched for cultural events, 24% followed cultural actors and 29% did not undertake any online activity connected with culture and cultural heritage [19, p. 11]. That shows that around 71% of the total European population having access to the Internet had undertaken some kind of activity connected to the cultural needs. At the same time, the data of cultural participation in Europe indicates that only 30% of EU-28 population take part in artistic activities on a regular (not on-line) basis. Among EU-28 residents aged 16 years or more—46% goes to the cinema, 43% attends live performances and 43% visits cultural sites [6, p. 124].

The popularity of the online access to culture is strongly prevailing among Europeans. Due to the Internet World statistics (June 2019) [12], nearly 88% of population living in European countries (not only limited to EU-28 countries) have access to the Internet. Moreover, the level of access to the Internet in Europe states 16% of the world’s Internet usage, while Europe has less than 11% of the world’s total population.

The effect of abandoning the offline culture participation to the benefit of the online participation should also be noted. Evidence suggests that only 1% of the Council of Europe citizens who participate in culture online do so without any real-world participation. Indeed, it seems that offline and online activity are complementary: those who participate more in any type of cultural activity are more likely to also use the Internet for cultural purposes [19, p. 12].

The digitalization of cultural heritage in Europe allows to widen the scope of culture participants as it provides for larger and possibly more diverse audience with opportunities to consume culture. This can help mitigate the underservicing of populations that would incur high costs for the non-virtual participation.

The COVID-19 outbreak forced the institutes the culture to face new challenges resulting from lockdowns in around 90 countries across the globe. The national governments required 3.9 billion of people (half of the world population) to stay home [4], while 40 countries in Europe introduced national lockdowns in various forms [22].

The lockdowns closed galleries, museums, cinemas, theatres and many other institutions across Europe. The situation triggered the immediate need to preserve access to their collections. The only possible way was to move with activity into the world wide web and create the online options of cultural activity.

The biggest European museums such as the British Museum, the Louvre Museum or the Hermitage Museum designed special online tours allowing visitors to see (on desktops of their computers) at least some parts of the collections with additional descriptions and multimedia instruments. The same applies to high number of galleries, as well as libraries, which opened their books’ collection to be accessed online [2].

Theatres and cinemas decided to carry on by offering online the plays (live or previously recorded) or movies including the premieres planned to happen when the pandemic broke out [31] (for example “Trolls World Tour” by DreamWorks Animation or “Lovebirds” by Paramount Pictures). The access is possible via designed for that purpose streaming platforms, mostly paid, but also through publicly open channels such as YouTube, where all the materials are accessible for free.

The biggest company providing worldwide information—Google has been widely engaged in access to culture activities. The Google Maps using the Google Street View tool opened possibility of virtual tours through the number of museums and galleries by using home computer. Moreover, the dedicated app—Google Arts & Culture allows people all around the world to maintain access to pieces of cultural heritage not only by just visiting museums but also by developing cultural taste and sensitivity. European examples in this sphere may include d’Orsay Museum in Paris or Rijksmuseum in Amsterdam. Of course, the mentioned developments are just the examples and had been known known already before the COVID-19 outbreak, however the possibility they gave to maintain the connection with culture during the compulsory “stay home” times is of the unique importance.

As access to cultural heritage is one of the human rights, the modern technologies replacing the traditional ways of experiencing culture allow not only to preserve those rights but to expand and strengthen their protection, when other human rights (such as right to liberty, freedom of assembly and association or freedom of religion) are often limited by the governments to minimalize the spread of pandemic. Such trend however, would need to be verified by further, detailed research after the pandemic ends.

For culture it is crucial to maintain contact with the audience as without audience culture cannot really exist and sometimes it is the audience that creates the culture itself. Maintaining contact with audience seems to be the most important reason for providing access to cultural heritage online. In addition, the cultural institutions, as institutions of all kind, do not want to be forgotten—people may lose the habit of visiting the specific cultural places and after reopening of the countries choose other places to spend time. The online contact helps to hold positive memory and, after the pandemic end, it will help to come back to the ordinary usage without losses in the audience. Another reason is cheering up people who are forced to stay home by governmental orders and helping them survive hard times by creating the ersatz of ordinary life.

The question to pose then is whether, after the pandemic and lockdowns end, the culture will stay online and what impact will it have on the offline activity. It may be imagined that part of the audience will stay online and never come back to the real cultural institutions as they will find the online experience sufficient. Others will come back to the offline experience, completely abandoning the online participation, whereas some will stay partly online and partly offline. The specific percentage of participants in each part is hard to define, but definitely will be interesting to observe and to carry out comprehensive research in the field.

## Conclusions

Authors of the paper believe that this brief study can be an interesting trend of transferring the widely understood culture broadcasters to the Internet sphere. The European perspective sets a firm example for the parts of the world where Internet is widely accessible and used. Of course, such trend had been visible (especially in the well Internet-connected regions) already before the pandemic started, but just after introducing the new governmental security measures it developed rapidly and took interesting directions. Currently, all most famous cultural institutions are active online, and a significant of the smaller ones also follows the path. The still vivid question is how the online culture participation will look like after the end of the pandemic.

The Council of Europe have been engaged in the protection of cultural rights for quite some time. Binding international conventions were signed in 1950s and even though the right to culture have never been as exposed as other, fundamental human rights, the European sensitivity for art and culture allowed to keep the right to culture as subject of the political and legal discussions resulting in adopted laws.

The ECtHR case-law clearly indicates that he right of access to online culture falls within the right of access to culture. The COVID-19 pandemic resulted in fact, that access to culture has been transferred to online dimension. This is a new situation and we can learn that protection of the right to culture should also be moved to the sphere of online activities.

The post-pandemic era will show how much of the transformation to the online cultural dimension is left with people at homes. Future cases in the ECtHR may bring answers to questions about protection mechanisms for the online access to cultural heritage. As the COVID-19 outbreak may not be the one-time global crisis and there are expectations for possible new virus developments, our online door to cultural treasures should be well protected and prepared for many challenges to come.

## Footnotes

1 The United Nations Special Rapporteur gives very similar standpoint when emphasizing: “by acting as a catalyst for individuals to exercise their right to freedom of opinion and expression, the Internet also facilitates the realization of a range of other human rights” [18, p. 6] including cultural and civil rights.

2 The United Nations Special Rapporteur on the promotion of the right to freedom of opinion and expression has declared that the Internet has become a key means by which individuals can exercise their right to freedom of opinion and expression, as guaranteed by the Universal Declaration of Human Rights and the International Covenant on Civil and Political Rights [10]. As this right includes cultural and civil rights—therefore it covers the access to culture and cultural heritage as well.
